# Supplementary material for: Weight stigma after bariatric surgery: A qualitative study with Brazilian women
Source: PLoS One. 2023 Jul 27;18(7):e0287822. doi: 10.1371/journal.pone.0287822 (PMC10374044; doi:10.1371/journal.pone.0287822)
Supplement: S1 File — In this supporting material we present the script that guided the semi-structured interviews. (DOCX) [file pone.0287822.s001.docx]

In this Supporting Informationwe present the script that guided the semi-structured interviews. Initially, Author 1 presented herself, explained the objectives of the interview and asked in the participant consented with the recording of the interview. After consent, the interview initiated with sociographic information, and then followed to questions that were relevant to the research

**Sociodemographic information**

- Please, can you tell me your full name?
- Please, can you tell me your age?
- Please, can you tell me the date of bariatric surgery?
- Please, can you tell me your height and current weight?
- Please, can you tell me what is your occupation?
- Please, can you tell me what is your schooling?
- Please, can you tell me which gender do you identify with?
- Please, can you tell me what is your sexual orientation?
- Please, can you tell me what is your marital status:
- Please, can you tell me how do you classify your skin color?
- Please, can you tell me with whom you live?
- Please, can you tell me your individual and family income? Who contributes the most to the family income?
- Please, can you tell me if you have children?

**Script of questions**

1. I would like to invite you to take a trip back in time. What are your childhood memories?
2. Do you remember what your body was like at that time? What was it like to deal with that body?
3. What about your eating habits, how were them like? Do you remember any food smells that you liked?
4. Do you remember having any health problems during this period? Did you look for a health professional (doctor, nutritionist, psychologist...) to follow up? How was this follow-up?
5. What were your childhood friendships like? Did you feel accepted in your group of friends?
6. Do you remember being bullied at this time? How was that?
7. Let’s move on to your adolescence, what memories do you have?
8. What was your body like as you grew up? If there were changes, what do you think led to them?
9. Was your eating habits similar in adolescence as it was in childhood or were there any changes? If yes, which ones?
10. Have your friendships stayed the same or changed? What were those friendships like? Did you feel accepted in your group of friends?
11. Do you remember being bullied at this time?
12. Did you have any romantic relationships as a teenager? Can you tell me how it went? If you didn’t have any relationship, did you have a reason for that?
13. Did you have any new health problems when you were a teenager? Did you look for a health professional (doctor, nutritionist, psychologist...) to follow up? How was this follow-up?
14. What do you like to do for fun? What are your interests? Was this like this before bariatric surgery?
15. Have your relationships changed as an adult? If yes, why?
16. What do you like most about your body and why? Was this like this before bariatric surgery?
17. What do you like least about your body and why? Was this like this before bariatric surgery?
18. Do you have any special care for your body? Was this like this before bariatric surgery?
19. How do you feel about your body these days? Are you happy or not with your current body? Why?
20. If you could change anything about your body before bariatric surgery, what would it be? And now?
21. How was the bariatric surgery process? That is, how was the decision to perform the procedure, how was the medical follow-up before the bariatric surgery? How did you feel in these accompaniments?
22. Was there any explanation about the bariatric surgery procedure? For example, did they explain the technique that would be used, what would be done, how the cut would be, etc.? How did you feel listening to these explanations?
23. What was your age and weight when you had bariatric surgery? What type of surgery was performed (sleeve, gastric bypass)?
24. What was the reaction of family and friends when they found out you were going to have the surgery? Did they participate in the decision or process in any way?
25. While waiting for bariatric surgery, did you prepare in any way or make any changes to your lifestyle? If yes, which ones? If not, what made you make that decision?
26. What is it like to be a person who has had bariatric surgery?
27. What do people tell you when you tell them you’ve had bariatric surgery?
28. Do you know other people who have had bariatric surgery? What do you think of the result they had with the surgery?
29. What does it mean to you to be “successful” in bariatric surgery? What do you think makes some people “successful” with surgery and others not? Do you consider your outcome a “success”?
30. In your opinion, do people who have had bariatric surgery eat differently from those who haven’t? If so, what’s the difference? There’s no right answer, I just want to know your opinion.
31. According to you, is someone who has had bariatric surgery viewed differently from someone who hasn’t? If yes, what’s the difference? Again, there is no right answer, I would just like to hear your opinion.
32. Did people have an opinion about your body and your diet before the surgery? And now? How is it for you?
33. Did your body hinder you in any way before the surgery? And now?
34. Have you ever done anything to change your body before bariatric surgery, such as going on a diet, starting some physical activity, taking any medication, other types of surgeries, even if they are not for weight loss? Did you make these attempts on your own or with professional supervision (and which professionals - doctors, nutritionists, psychologists, physical educators)? What and how were these experiences?
35. How do you deal with your food today? What do you think of it? For example, who cooks, how is food organized in your house, who does the shopping (before and after the pandemic)?
36. About the post-operative food, who made the food for you? Was there any care with this food?
37. Do you do physical activity nowadays? Has it always been like this?
38. What is more difficult and what changes the most after bariatric surgery?
39. Do you think the changes in your body were related to changes in your life (such as changes in work, friendships, relationships)?
40. Did you have complications after bariatric surgery? How was that?
41. After having the bariatric procedure, did you continue to have any health problems you had before? Have any new health problems popped up?
42. Do you follow up with health professionals (doctors, nutritionists, psychologists, physical educators, etc.) after bariatric surgery? Do you notice differences in this service before and after bariatric surgery?
43. Do you have any fears these days?
44. What does it mean to gain weight for those who have had bariatric surgery?
45. Would you recommend the surgery to others?
46. Do you think the pandemic affected you? Did it affect the outcome of your surgery? As?
47. Is there anything you would like to add, is there anything I didn’t ask you that you would like to talk about?
