# Supplementary material for: Weight stigma after bariatric surgery: A qualitative study with Brazilian women
Source: PLoS One. 2023 Jul 27;18(7):e0287822. doi: 10.1371/journal.pone.0287822 (PMC10374044; doi:10.1371/journal.pone.0287822)
Supplement: Attachment — Submitted filename: Response to Reviewers.docx [file pone.0287822.s003.docx]

Dear Editor Professor Dra. Emily Chenette:

We are pleased to re-submit the revised version of the manuscript “If the weight is gone, is the stigma also gone? A qualitative study of the perceptions and experiences of Brazilian older and younger women who underwent bariatric surgery”. We highlight that after the reviewers’ comments, the title has changed to “A qualitative study of Brazilian women’s perceptions and experiences of weight stigma after bariatric surgery”, as suggested. We have extensively revised the manuscript according to the suggestions made by the referee and the editor. An item-by-item response is presented below. All changes in the manuscript are highlighted with tracked changes. We hope that these changes will meet with your approval.

**- Editor:**

**Article formatting**

Editor comment: Please ensure that your manuscript meets PLOS ONE’s style requirements, including those for file naming. The PLOS ONE style templates can be found at https://journals.plos.org/plosone/s/file?id=wjVg/PLOSOne_formatting_sample_main_body.pdf and

https://journals.plos.org/plosone/s/file?id=ba62/PLOSOne_formatting_sample_title_authors_affiliations.pdf

Response: We have formatted the manuscript accordingly.

**Methods**

Editor comment: Please provide additional details regarding participant consent. In the ethics statement in the Methods and online submission information, please ensure that you have specified whether: 1) whether the ethics committee approved the verbal/oral consent procedure, 2) why written consent could not be obtained, and 3) how verbal/oral consent was recorded. If your study included minors, please state whether you obtained consent from parents or guardians in these cases. If the need for consent was waived by the ethics committee, please include this information.

Response: We have clarified these aspects in the Methods section and in the online submission information. Regarding the question about why written consent could not be obtained, we conducted the research during the pandemic, and therefore, the interviews were conducted remotely via WhatsApp video calls. Asking the participants for a written consent could be demanding for them, and possibly, excluding. For example, as they would have to print, then scan the signed document and finally send to Author 1, some of them, especially the older women, could have difficulty to do so. Thus, we understood that both oral consent and digitally recorded informed consent would provide access to all participants.

**Data availability**

Editor comment: In your Data Availability statement, you have not specified where the minimal data set underlying the results described in your manuscript can be found. PLOS defines a study’s minimal data set as the underlying data used to reach the conclusions drawn in the manuscript and any additional data required to replicate the reported study findings in their entirety. All PLOS journals require that the minimal data set be made fully available. For more information about our data policy, please see http://journals.plos.org/plosone/s/data-availability.

“Upon re-submitting your revised manuscript, please upload your study’s minimal underlying data set as either Supporting Information files or to a stable, public repository and include the relevant URLs, DOIs, or accession numbers within your revised cover letter. For a list of acceptable repositories, please see http://journals.plos.org/plosone/s/data-availability#loc-recommended-repositories. Any potentially identifying patient information must be fully anonymized.

Important: If there are ethical or legal restrictions to sharing your data publicly, please explain these restrictions in detail. Please see our guidelines for more information on what we consider unacceptable restrictions to publicly sharing data: http://journals.plos.org/plosone/s/data-availability#loc-unacceptable-data-access-restrictions. Note that it is not acceptable for the authors to be the sole named individuals responsible for ensuring data access.

We will update your Data Availability statement to reflect the information you provide in your cover letter.

Response: We have included or data set, which is available in a public repository from the University of São Paulo, available at: https://repositorio.uspdigital.usp.br/handle/item/399

**General comments**

Editor comment: The manuscript focuses on a relevant area for research and their results are relevant. There are, however, some flaws that must be corrected. Special attention must be directed to Reviewer #2 comments on the use of stigmatizing language and the “visual analysis” mentioned in lines #208-2015. Methodology section should be improved, including Reviewer #1 recommendation of better description of the characteristics of healthcare and follow up of study participants. The text also requires extensive rewriting to achieve appropriate standards of readability.

Response: We have addressed the aspects mentioned and they are fully addressed in the responses below.

**- Reviewer’s Responses to Questions**

Comments to the Author

Reviewers’ comments: 1. Is the manuscript technically sound, and do the data support the conclusions?

The manuscript must describe a technically sound piece of scientific research with data that supports the conclusions. Experiments must have been conducted rigorously, with appropriate controls, replication, and sample sizes. The conclusions must be drawn appropriately based on the data presented.

Reviewer #1: Yes

Reviewer #2: Partly

Response: We have extensively reviewed the manuscript to address these matters.

________________________________________

2. Has the statistical analysis been performed appropriately and rigorously?

Reviewer #1: N/A

Reviewer #2: N/A

________________________________________

3. Have the authors made all data underlying the findings in their manuscript fully available?

The PLOS Data policy requires authors to make all data underlying the findings described in their manuscript fully available without restriction, with rare exception (please refer to the Data Availability Statement in the manuscript PDF file). The data should be provided as part of the manuscript or its supporting information, or deposited to a public repository. For example, in addition to summary statistics, the data points behind means, medians and variance measures should be available. If there are restrictions on publicly sharing data—e.g. participant privacy or use of data from a third party—those must be specified.

Reviewer #1: No

Reviewer #2: No

Response: We have included or data set, which is available in a public repository from the University of São Paulo, available at: https://repositorio.uspdigital.usp.br/handle/item/399

________________________________________

4. Is the manuscript presented in an intelligible fashion and written in standard English?

PLOS ONE does not copyedit accepted manuscripts, so the language in submitted articles must be clear, correct, and unambiguous. Any typographical or grammatical errors should be corrected at revision, so please note any specific errors here.

Reviewer #1: Yes

Reviewer #2: No

Response: The manuscript was submitted to an extensive English revision by native English speakers.

**- Reviewer 1**

**Introduction**

Reviewer comment: Line 72-72 – “In Brazil, bariatric procedures have been available at no cost to patients since 1999 73 through the Unified Public Health System or the Sistema Único de Saúde [10]” but even so, it is necessary to consider how is the access to this intervention, as the demand for the procedure is certainly very high. Clarify regional differences and the issue of equity.

Response: We have addressed these aspects in the Introduction section.

**Methods**

Reviewer comment: The authors inform that patients being followed by the Bariatric Surgery Outpatient Clinics of HCFMUSP. Describe better how women get there, referral criteria and how they are followed up, how often and for how long is the follow-up, before/after surgery, and by which professionals.

Response: We have these aspects in the Methods section.

**Methods**

Reviewer comment: The script used for the interviews was not presented, which guiding questions and the previous experience of the researcher (Author1) was not clarified for carrying out both the interviews and data analysis - this aspect is very relevant for the quality of qualitative research.

Response: In the reviewed version of the manuscript, we included the script that guided the interviews (Supporting Information 1). Also, in the Methods section, we included the previous experience and training of Author 1 regarding conducted individual interviews.

**Results**

Reviewer comment: On Table 1, complete your title indicating location/year. And explain how this information on the women’s characteristics was obtained, whether directly with the women or clinic provided, from a medical record.

Response: We completed the title indicating location and year. The information on the women’s characteristics was obtained directly with the participants, during the individual interviews. We added this information in the Results section. These questions can be seen in the script that guided the interviews, submitted as a Supplementary Material.

**Discussion**

Reviewer comment: In the lines 650-651 the authors indicate “Although our participants also had pre-operative support groups, this support may have been insufficient to combat stressful social messaging.” This suggests that there is no further follow-up, but the women were recruited at this service after the surgery.

Response: We have clarified this aspect in the Discussion section.

**- Reviewer 2**

**General**

Reviewer comment: Can you explain if and how you included patient and public involvement in this work? For example, what stages were they involved and what activities/input did they have.

Response: Participants were patients who underwent bariatric surgery at Hospital das Clínicas of the School of Medicine, University of São Paulo. Their involvement with the research included the individual semi-structured interviews. They were contacted by Author 1 and, if they agreed to participate, the interview was scheduled. These procedures are extensively described in the Methods section.

**General**

Reviewer comment: Throughout the manuscript, there are quantifying words or sentences for example: ‘For all participants’ or ‘Some of the women’. Please delete the quantifying words.

Response: These changes were made accordingly.

**General**

Reviewer comment: The manuscript would benefit greatly from a review of the grammar/scientific writing.

Response: The manuscript was submitted to an extensive English revision by native English speakers.

**Title**

Reviewer comment: The title would stand alone better, be more grammatically correct, and be clearer without the question. For example, as “A qualitative study of Brazilian women’s’ perceptions and experiences of weight stigma after bariatric surgery”. I echo the same point for the short title. Perhaps a reframe to “A qualitative study of stigma after bariatric surgery”.

Response: The changes in the Title were made accordingly.

**Abstract**

Reviewer comment: Can you please reframe the following into a research aim/objective (rather than a question): “However, how do bariatric patients perceive and internalize this stigma in different life stages? Specifically, is older age a risk factor?”. The current framing is a little too colloquial. Can you explicitly state the method of data analysis used? E.g., was it thematic analysis or something different? The abstract is missing a conclusion. Can you please add a sentence concluding your findings, and perhaps suggesting how the findings may be used in the future? You could make your results more concise if you need more word count to allow this.

Response: The changes in the Abstract were made accordingly.

**Introduction**

Reviewer comment: Can the tone of the following sentence be softened: “all of which induce severe weight loss through the surgical reduction of the stomach and/or intestines”. For example, to “all of which aim to induce severe weight loss through the surgical reduction of the stomach and/or intestines”

Response: The change was made accordingly.

**Introduction**

Reviewer comment: Please add references to support: “Bariatric patients often present for surgery with a history of complex anxieties and negative social experiences stemming from weight-related stigma.”

Response: We added the following references: 1) da Silva SSP and da Costa Maia Â (2012) Obesity and treatment meanings in bariatric surgery candidates: a qualitative study. Obesity surgery 22(11), 1714-1722. https://doi.org/10.1007/s11695-012-0716-y; 2) Trainer S, Brewis, A and Wutich A (2017) Not ‘taking the easy way out’: reframing bariatric surgery from low-effort weight loss to hard work. Anthropology & Medicine 24(1), 96-110. https://doi.org/10.1080/13648470.2016.1249339; 3) Hansen, B and Dye, MH (2018) Damned if You Do, Damned if You Don’t: The Stigma of Weight Loss Surgery, Deviant Behavior, 39:2, 137-147, DOI: 10.1080/01639625.2016.1263081

**Introduction**

Reviewer comment: The word ‘deviant’ isn’t necessary to the message in the sentence on line 82 in the second paragraph of the introduction – can you please remove.

Response: The change was made accordingly.

**Introduction**

Reviewer comment: In the definition of weight stigma by Brewis, stigmatising language itself is used (“being overweight or obese”). Can you please reframe this to move away from this language and use person-first language instead?

Response: This was a direct citation of the term that the Brewis used. Nonetheless, we understand the need to change, and we have done such change as the following: *“Following Brewis [16] we define weight stigma as moral discrediting that people experience from others or apply to themselves because of the negative social meanings attached to the size of bodies”*.

**Introduction**

Reviewer comment: In the introduction (and manuscript) you move between using the term weight stigma and weight-related stigma. Can you please amend the manuscript so you use consistent language throughout?

Response: The change was made accordingly and used the term “weight stigma” throughout the manuscript.

**Introduction**

Reviewer comment: In the introduction, you use the stigmatising language of “fat”. Can you please address this throughout the manuscript to use person-first language?

Response: The change was made accordingly. We just kept the word “fat” throughout the manuscript when it was an *emic* word or when referring to authors that employed this word.

**Introduction**

Reviewer comment: The introduction is very lengthy and consistently takes multiple paragraphs to make a point that could be made more clearly in one paragraph. Could you please review the introduction to see where content could be made more concise, and therefore clearer for the reader to follow?

Response: The change was made accordingly.

**Introduction**

Reviewer comment: Can you soften the tone in the following sentence: “Weight loss induced by surgical intervention is so rapid that it produces a great deal of excess skin and it also begins at the “ends,” i.e., people tend to lose weight in their heads, arms, and legs before they lose it in their torsos”. For example, to: “Weight loss induced by surgical intervention is so rapid that it often produces a great deal of excess skin. Furthermore, people tend to lose weight in their heads, arms, and legs before they lose it in their torsos, influencing their body shape and size”.

Response: The change was made accordingly.

**Introduction**

Reviewer comment: Despite the length of the introduction, it does not note the potential implications of addressing the research aim. For example, how will this inform public health?

Response: The change was made accordingly.

**Methods**

Reviewer comment: Can you please move the ethical approval statement to the start of the methods section?

Response: The change was made accordingly.

**Methods**

Reviewer comment: Overall, there is a great lack of detail in the data analysis section. Please review and add more detail here to aid the reader to thoroughly understand what you have done with enough detail for them to replicate the processes. For example: You note participants who fit the ‘proposed profile’ were invited to interview - what is the proposed profile? What sampling method did you use? Did you use purposeful sampling, snowball, opportunist? How were the interviews transcribed? Was this by an external company or by the lead author?

Response: The change was made accordingly.

**Methods**

Reviewer comment: You note in the data collection section that “This two-encounters approach proved to be quite effective in obtaining data and resulted in the chance to complete the interview protocol in a way that was practical and feasible for the participants’ schedules”. I’m unclear how conducting two interviews would be more practical and feasible for someone’s schedule than conducting one interview – can you explain? Also, “proved to be quite effective in obtaining data” is very unclear and non-specific. I’m not sure the value this sentence brings or the message you are trying to communicate.

Response: Our interviews were long, lasting an average of three hours or more. When engaging with the participants, Author 1 exposed the estimate time of the interview and the participants asked to have the interview divided in two encounters, and sometimes, a third encounter was necessary. This was because it was much more feasible to them to provide Author 1 two (or three) shorter encounters than one longer single encounter. In that sense, if we insisted on having the interview in one single encounter, most of the participants could feel discouraged to participate. Therefore, this is what we meant by this proving “to be quite effective in obtaining data”.

**Methods**

Reviewer comment: You state that “Notably, the time gap between the first and second interviews led to increased reflexivity among the participants and the researcher, who reviewed the recording of the first encounter to note gaps that needed to be covered in the second.” Reflexivity is the acknowledgement of your role and influence on the research process, so it does not make sense in this sentence. Perhaps you were meaning data quality? I’d also suggest softening the tone of many of these sentences. You cannot be certain that the time gap you had led to increased (presumably) data quality, but you could say that was the aim/purpose of the time gap.

Response: The change was made accordingly.

**Methods**

Reviewer comment: I strongly oppose to many of the messages from line 208 to 215, and feel the messages themselves exacerbate the stigmatisation of those living with overweight or obesity. If you are analysing participants experiences (e.g., feeling like they had not lost enough weight), then it is not your role and it is inappropriate to say that you must visually assess/observe their bodies to validate this. Furthermore, the notation of having to “rely on her description” is highly problematic. If you are analysing someone’s experiences, then the analysis and resultant publication should be aiming to represent the voices of the participant, not your assessment as to whether these experiences are reliable. Please remove this paragraph.

Response: We removed this paragraph.

**Methods**

Reviewer comment: Lines 227 to 229 are hard to follow – can you please rephrase?

Response: We removed this paragraph.

**Methods**

Reviewer comment: In lines 228-229, you note themes as emerging as significant. These is vast amounts of literature noting themes as “emergent” to be problematic as it suggests that the themes always existed within the data, and voids the influence of our positionality as the researcher on the process. Can you please review this throughout the manuscript to remove this concept?

Response: We reviewed the manuscript and removed this concept as requested. Also, we clarified about the development of themes in the Data Analysis subsection.

**Methods**

Reviewer comment: In lines 231-233, you state that you identified excerpts of interviews relevant to the themes of interest, but you have not explained the process of theme development at this stage. How did you develop the themes of interest?

Response: We have clarified the data analysis, as well as the development of themes. It is in the Data Analysis subsection.

**Methods**

Reviewer comment: You have not described how the interview schedule was produced or referenced to a copy of the interview schedule in the supplements. Please add these details.

Response: We have included a copy of the interview schedule in the Supporting Information. We clarify that we are presenting both the interview script and schedule as Supporting Informations.

**Methods**

Reviewer comment: There lacks a section on the researcher positionality and how they managed the impact of this through reflexivity. Please can the authors address this.

Response: We have addressed these aspects accordingly at the end of Discussion section.

**Results**

Reviewer comment: In Table 1, please remove the percent and standard deviation. These are not generally used in qualitative studies.

Response: We removed the percent and standard deviation from Table 1 accordingly.

**Results**

Reviewer comment: You presented quotations in multiple languages, but have not noted this translation process in the methods section. Please add this information.

Response: We have added this information accordingly.

**Results**

Reviewer comment: Throughout the results section, you state findings in affirmative language – for example, “she asked if this man had…”. A better/more accurate way of describing the findings is to include acknowledgement of the interpretation of their accounts by including prefaces – for example, such as “the participant described/expressed that she asked the man...”. It would strengthen the manuscript to review and revise these areas.

Response: We have reviewed and revised these areas accordingly.

**Results**

Reviewer comment: The final lines of the first theme (lines 314 to 317) don’t seem to fit with the narrative of the rest of the theme. Can you please review and either remove or clarify their fit with the narrative?

Response: We have removed these lines.

**Results**

Reviewer comment: Line 323 – are these self-appointed by the participant or by their peers?

Response: The comments were expressed by other people. We changed the text to clarify the message.

**Results**

Reviewer comment: Between lines 329 to 335, you describe others having concerns over the participants health. Could you please include a supporting quotation here?

Response: We included supporting quotations in this paragraph.

**Results**

Reviewer comment: On lines 348 to 350, you introduce new literature within the results section. You shouldn’t incorporate other literature in this section so could you remove and rephrase this section. For example, you could simply say “Participants who described experiencing small weight regain after weight loss described being reprimanded by others”.

Response: We removed citation and made the change in the paragraph as suggested.

**Results**

Reviewer comment: Line 360 – is “melt off” a quote from a participant or your own writing? If a quote, can quotations marks please be added. If it is your own writing, can it be rephrased to be more in line with the language and writing style of the piece.

Response: The use of the word “melt off” was a choice of the Authors. Nonetheless, we agree that it was not in line with the language and writing style of the manuscript and have changed it accordingly.

**Results**

Reviewer comment: Again in lines 366 to 370, you introduce new literature and compare findings to existing evidence. Please remove this from the results. This is repeated in areas throughout the results section – can you please review this throughout.

Response: We removed lines 366 to 370 from the results and revised the entire section to remove other citations that were previously included.

**Results**

Reviewer comment: Lines 391 to 392 begin to discuss the results, rather than present the results. Can you please move this to the discussion or remove?

Response: We removed lines 391 to 392 as requested.

**Results**

Reviewer comment: Line 393 “aggressive” and line 396 “mad”– were these words used by the participants or is this your own language choice?

Response: We changed these lines to clarify that these words were used by the participants.

**Results**

Reviewer comment: Line 400 to 401 – did participants say that the advice was not backed up by evidence, or is this your own commentary? If the latter, please move to the discussion or remove.

Response: We removed lines 400 to 401 as requested.

**Results**

Reviewer comment: Line 423 – did the participant describe the foods as “fattening foods” themselves? If so, can you please add quotation marks. If not, can you please remove/reframe this concept.

Response: We have clarified line 423 to show that this was how the participants described the foods and included a supporting quotation.

**Results**

Reviewer comment: Lines 444 to 450 do not belong in the results section altogether, and the key points should only remain in the introduction. I recommend to remove altogether and keep the focus to the findings of this particular study.

Response: We removed lines 444 to 450 as requested.

**Results**

Reviewer comment: Lines 470 to 472 – I recommend to rephrase these lines. Currently, this reads as your perception rather than a reflection of participants voices and experiences.

Response: We have removed lines 470 to 472 as requested.

**Results**

Reviewer comment: Unit of meaning 4 – this title includes non-person first language. Can this please be amended.

Response: We have included person-first language, except when the participant herself voiced a non-person first language, such as “fat” (in Brazilian Portuguese, “gorda”).

**Results**

Reviewer comment: Unit of meaning 4 does not appear to have a well-bounded message or narrative. The points within feel quite distinct from one another at times, and they do not always relate to the overarching title given to the theme. I think this theme needs a reworking entirely to reclarify what the key messages are, and refocused the text to make those clearer.

Response: We have reworked on this theme and changed its title to clarify the message.

**Results**

Reviewer comment: Overall, the themes are extremely long which makes it difficult at times to follow the key points – they can get lost in the length of text. Can you review the themes and identify where things could be cut down or made more concise? For example, when a message is communicated, you often include many examples and quotations, and this many are not always necessary to support and evidence the message communicated. You could look to cut down these areas. In addition, much of the themes feels like a reproduction of the contributing interviews, rather than a summative/themed narrative cutting across the interviews, thus contributing to the length of the themes and the dilution of the messages within.

Response: We have extensively revised the Results section, cut down some information and made it more concise.

**Results**

Reviewer comment: A key aim of the paper was to compare the differences in experiences of younger and older women, but from how the results are presented (i.e., experiences of each group intertwined throughout, without much comparative analytical commentary) it is not possible to do this. Have you considered separately the results in each theme by younger and older to make clearer distinctions of these experiences?

Response: As suggested, we have separated the results in each theme by: 1) Common experiences among the younger and older participants and; 2) The experiences of the older participants.

**Discussion**

Reviewer comment: Lines 561-562. In the introduction, the aim is framed to compare younger and older women, rather than to focused explicitly on the experiences of older women as it is framed in this line. Can you please amend this line to be consistent?

Response: The change was made accordingly.

**Discussion**

Reviewer comment: Paragraph 2 (lines 576 to 589) mostly represents the findings of the study, and there is limited comparison to other available literature. It is also a little to strong to claim this finding to be ‘novel’ due to perceived strength of messaging, and I recommend softening the tone here. In addition, the end of the paragraph lacks cited literature to support the statements made (i.e., lines 586-589). The language/grammar would benefit from reviewing in this area too.

Response: We have expanded the comparison to other available literature in Paragraph 2 and softened the tone regarding the novel finding. Finally, the manuscript was submitted to an extensive English revision by native English speakers.

**Discussion**

Reviewer comment: Consistently throughout the discussion, the paragraphs tend to reproduce the findings of the study, with very limited contextualisation or comparison with the wider literature base.

Response: We revisited the Discussion and made extensive changes, as well as included more contextualization or comparison with the wider literature base.

**Discussion**

Reviewer comment: Lines 620 to 624 – this sentence is extremely long and hard to follow, and it also introduces several new, large points (e.g., Foucault’s notion of biopower). These concepts should be introduced in the introduction if they are to be discussed because, as currently written, this section does not give enough context or background information for the reader to fully conceptualise the messages intended.

Response: To attend the following comment of the Reviewer, we have removed Lines 620 to 634.

**Discussion**

Reviewer comment: Paragraph on lines 620 to 634 does not seem to fit with the narrative of the entire manuscript, and the messages made feel extremely distinct and off-topic. I’m unclear how your findings compare to this and fit within these messages. In addition, in areas, it reads to have been influenced by opinions rather than a balanced discussion of this complex area. For example, the description of weight management to be “punitive” and “authoritarian” and aligned with assumptions that “those with a higher body weight lack knowledge and understanding about health practices, make poor choices, and refuse to take responsibility for their health and well-being”. If this section is to remain, it would require reworking to provide a scientifically critical and balanced discussion from multiple lens.

Response: We have removed Lines 620 to 634.

**Discussion**

Reviewer comment: Line 631 – we do not tend to gender the authors of literature we are citing as we cannot assume one’s gender. Can you please review this throughout the manuscript.

Response: We believe it is essential to point out that all the authors who sign this article stand out for investigating the different social markers of difference, including gender and sexuality, from an intersectional perspective. In this sense, the assumption of a particular gender is far from our perspective. If we de-gender an author, we would use the pronouns he and him, which are already gendered. Therefore, we decided to continue to refer to the authors as they refer to themselves as a gender through their productions and first names. We believe that de-gendering authors, without their consent, could become a lack of respect for their gender ascriptions.

**Discussion**

Reviewer comment: The manuscript lacks a section highlighting the limitations of the study. Please review and add this to the manuscript. This should include (among others) – the impact of researcher positionality and the recall bias due to the long time span between the surgeries of participants.

Response: We have included a section highlighting the limitations of the study as requested.

**Conclusion**

Reviewer comment: Lines 679-682, and lines 686-687. Insufficient comparison between the groups has been presented through the manuscript as currently written to make a clear conclusion about the differences in experiences. I would recommend reworking the results section to make this potentially possible to conclude, or remove these conclusions.

Response: We changed the focus of the article to do a description of older and younger women after bariatric surgery. We also have separated the results in each theme by younger and older to make clearer distinctions of these experiences (as highlighted in a response above). With these changes, we believe that the Conclusion is now sufficient support the data presented.

We thank you again for the valuable contributions of the reviewer and yourself and for the opportunity to submit our manuscript for your consideration.

Kind regards,

The authors.
