# Supplementary material for: Weight stigma after bariatric surgery: A qualitative study with Brazilian women
Source: PLoS One. 2023 Jul 27;18(7):e0287822. doi: 10.1371/journal.pone.0287822 (PMC10374044; doi:10.1371/journal.pone.0287822)
Supplement: Attachment — Submitted filename: Stigma qual review (R2). docx.docx [file pone.0287822.s004.docx]

Thank you for your responses to my comments - the article is reading much better. To note - your responses should direct the reviewer to the page and line numbers where the edits in text have been made and, if possible, provide the edited text within your response also. It makes the process of peer review substantially more time consuming and difficult to not be given direction or detail within responses.

Original reviewer comment: Can you explain if and how you included patient and public involvement in this work? For example, what stages were they involved and what activities/input did they have.

Author response: Participants were patients who underwent bariatric surgery at Hospital das Clínicas of the School of Medicine, University of São Paulo. Their involvement with the research included the individual semi-structured interviews. They were contacted by Author 1 and, if they agreed to participate, the interview was scheduled. These procedures are extensively described in the Methods section.

Reviewer response: Patient and public involvement (PPI) is an active partnership between members of the public and researchers, where the public are involved in aspects of the study/research (such as informing the study design, supporting with analysis). The participants of the study do not count as PPI. You can read more about this on: https://www.rds-sc.nihr.ac.uk/ppi-information-resources/ Can you please provide a response given this explanation of PPI.

Original reviewer comment: Can you please reframe the following into a research aim/objective (rather than a question): “However, how do bariatric patients perceive and internalize this stigma in different life stages? Specifically, is older age a risk factor?”. The current framing is a little too colloquial. Can you explicitly state the method of data analysis used? E.g., was it thematic analysis or something different? The abstract is missing a conclusion. Can you please add a sentence concluding your findings, and perhaps suggesting how the findings may be used in the future? You could make your results more concise if you need more word count to allow this.

Author response: The changes in the Abstract were made accordingly.

Reviewer response: You have not added the method of data analysis used to the abstract – please address this. The conclusion added goes beyond what your findings can support, and should be softened in tone to be more appropriate to the levels of findings. Specifically, the findings do not inform a targeted approach, however they might suggest future research should explore whether a targeted approach might be more effective.

Original reviewer comment: In the introduction, you use the stigmatising language of “fat”. Can you please address this throughout the manuscript to use person-first language?

Author response: The change was made accordingly. We just kept the word “fat throughout the manuscript when it was an emic word or when referring to authors that employed this word.

Reviewer response: Can you please edit the text to have quotation marks (or something similar) to make it clear to the reader than the remaining use of the word ‘fat’ is not your words, but quoting participants etc.

Original reviewer comment: Line 631 – we do not tend to gender the authors of literature we are citing as we cannot assume one’s gender. Can you please review this throughout the manuscript.

Authors response: We believe it is essential to point out that all the authors who sign this article stand out for investigating the different social markers of difference, including gender and sexuality, from an intersectional perspective. In this sense, the assumption of a particular gender is far from our perspective. If we de-gender an author, we would use the pronouns he and him, which are already gendered. Therefore, we decided to continue to refer to the authors as they refer to themselves as a gender through their productions and first names. We believe that de-gendering authors, without their consent, could become a lack of respect for their gender ascriptions.

Reviewer response: From viewing the submitted manuscript, I see that this section has now been deleted. Regardless, it is important to clarify this point. Not assuming gender wouldn’t ever result in the automatic use of he/him – if pronouns were ever essential to be used, the pronouns used within English written language are they/them when not assuming gender. In addition, you cannot be sure of a person’s gender from their first name – this bears no reflection of gender. I’m unsure what the authors mean when they say ‘their productions’. Generally, ‘the authors found’ or ‘the study found’ is used instead of gendered terms – this is the practice you have used elsewhere in this article.
