# Supplementary material for: Weight stigma after bariatric surgery: A qualitative study with Brazilian women
Source: PLoS One. 2023 Jul 27;18(7):e0287822. doi: 10.1371/journal.pone.0287822 (PMC10374044; doi:10.1371/journal.pone.0287822)
Supplement: Attachment — Submitted filename: Response to reviewers (R2) 13.06.23.docx [file pone.0287822.s005.docx]

Dear Editor Professor Dr. Emily Chenette:

We are pleased to re-submit the revised version of the manuscript “Weight stigma after bariatric surgery: A qualitative study with Brazilian women”, as suggested. We have extensively revised the manuscript according to the suggestions made by the referee and the editor. An item-by-item response is presented below. All changes in the manuscript are highlighted with tracked changes. We hope that these changes will meet with your approval.

**- Editor:**

**Introduction**

Editor comment: Some references need to be added/edited in the introduction section.

Response: We have corrected the references accordingly.

**Methods**

Editor comment: In the research setting and study population section, the eligibility criteria includes gendered language (i.e., her). I assume men and women would be eligible for bariatric surgery, therefore referring to ‘her’ only is not appropriate.

Response: We have changed that as requested (page 7, lines 181, 182).

**General comment**

The full paper requires English language editing, engaging an editing service may support this. There are many long and complex sentences that need refining also.

Response: Three native English speakers have revised independently the text to address these questions. The changes regarding English language editing were not highlighted throughout the manuscript.

**- Reviewer 1**

**Abstract**

Original reviewer comment (R1): Can you please reframe the following into a research aim/objective (rather than a question): “However, how do bariatric patients perceive and internalize this stigma in different life stages? Specifically, is older age a risk factor?”. The current framing is a little too colloquial. Can you explicitly state the method of data analysis used? E.g., was it thematic analysis or something different? The abstract is missing a conclusion. Can you please add a sentence concluding your findings, and perhaps suggesting how the findings may be used in the future? You could make your results more concise if you need more word count to allow this.

Author response (R1): The changes in the Abstract were made accordingly.

Reviewer response (R2): You have not added the method of data analysis used to the abstract – please address this. The conclusion added goes beyond what your findings can support, and should be softened in tone to be more appropriate to the levels of findings. Specifically, the findings do not inform a targeted approach, however they might suggest future research should explore whether a targeted approach might be more effective.

Response (R2): The method of data analysis was informed as the following (page 2 lines 42-43): **“The resulting text was then analyzed using thematic analysis”**. To clarify, thematic analysis was the method used for data analysis. Regarding the conclusions, we have changed as suggested. In the previous version, it was as the following: “Our study can thus inform a more targeted approach to care for different cohorts who undergo bariatric surgery, an approach that would emphasize the importance of developing coping strategies with respect to experiences of stigma and discrimination after surgery”. We have changed in the revised version of the manuscript to (page 2, lines 53-56): **“Our study suggest future research should explore whether a targeted approach might be more effective, for example, an approach that would emphasize the importance of developing coping strategies with respect to experiences of stigma and discrimination after surgery.”** We have also added this information in other sections of the manuscript (i.e., at the end of Introduction and at the end of Conclusions).

**Introduction**

Original reviewer comment (R1): In the introduction, you use the stigmatising language of “fat”. Can you please address this throughout the manuscript to use person-first language?

Author response (R1): The change was made accordingly. We just kept the word “fat throughout the manuscript when it was an emic word or when referring to authors that employed this word.

Reviewer response (R2): Can you please edit the text to have quotation marks (or something similar) to make it clear to the reader than the remaining use of the word ‘fat’ is not your words, but quoting participants etc.

Response (R2): We have added quotation marks to make it clear to the reader that the remaining use of the word ‘fat’ is not our words, but quoting participants or authors that employed this word.

**Methods**

Original reviewer comment (R1): Can you explain if and how you included patient and public involvement in this work? For example, what stages were they involved and what activities/input did they have.

Author response (R1): Participants were patients who underwent bariatric surgery at Hospital das Clínicas of the School of Medicine, University of São Paulo. Their involvement with the research included the individual semi-structured interviews. They were contacted by Author 1 and, if they agreed to participate, the interview was scheduled. These procedures are extensively described in the Methods section.

Reviewer response (R2): Patient and public involvement (PPI) is an active partnership between members of the public and researchers, where the public are involved in aspects of the study/research (such as informing the study design, supporting with analysis). The participants of the study do not count as PPI. You can read more about this on: https://www.rds-sc.nihr.ac.uk/ppi-information-resources/ Can you please provide a response given this explanation of PPI.

Response (R2): Thank you for your comment. We have not understood that this was the aim of your question, we apologize for that. We have clarified the Patient and Public involvement as the following (novel information is in **bold**), in the Methods section (page 8, lines 208-212): “In the initial contact, Author 1 introduced herself, explained why she was contacting them, the purpose of the research, and clarified that if they agreed to participate, the participation would be voluntary, and the findings would be kept confidential. **This study does not qualify as Patient and Public Involvement (PPI) research, because PPI refers to an active partnership between members of the public and researchers, in which members of the public work alongside the research team and are actively involved in contributing to the research process as advisers and possibly as co-researchers. This did not happen in our study (NIHR, 2023).**

**General comment**

Original reviewer comment (R1): Line 631 – we do not tend to gender the authors of literature we are citing as we cannot assume one’s gender. Can you please review this throughout the manuscript.

Authors response (R1): We believe it is essential to point out that all the authors who sign this article stand out for investigating the different social markers of difference, including gender and sexuality, from an intersectional perspective. In this sense, the assumption of a particular gender is far from our perspective. If we de-gender an author, we would use the pronouns he and him, which are already gendered. Therefore, we decided to continue to refer to the authors as they refer to themselves as a gender through their productions and first names. We believe that de-gendering authors, without their consent, could become a lack of respect for their gender ascriptions.

Reviewer response (R2): From viewing the submitted manuscript, I see that this section has now been deleted. Regardless, it is important to clarify this point. Not assuming gender wouldn’t ever result in the automatic use of he/him – if pronouns were ever essential to be used, the pronouns used within English written language are they/them when not assuming gender. In addition, you cannot be sure of a person’s gender from their first name – this bears no reflection of gender. I’m unsure what the authors mean when they say ‘their productions’. Generally, ‘the authors found’ or ‘the study found’ is used instead of gendered terms – this is the practice you have used elsewhere in this article.

Response (R2): We thank you for your clarification. If the section was in the manuscript, we would certainly change it according to your recommendations.

We thank you again for the valuable contributions of the reviewer and yourself and for the opportunity to submit our manuscript for your consideration.

Kind regards,

The authors.
